# Bioactive Compounds from Agro-Industrial By-Products: Green Recovery Technologies, Analytical Characterization, and Industrial Applications

**DOI:** 10.3390/foods15132406

**Published:** 2026-07-07

**Authors:** Jessica J. Hurtado-Rios, Yenizey M. Alvarez-Cisneros, Héctor Escalona-Buendía, Carmen G. Hernández-Valencia, María de Lourdes Pérez-Chabela, María Aurora Pintor-Jardines, Jorge Soriano-Santos, Gloria Maribel Trejo-Aguilar, Edith Ponce-Alquicira

**Affiliations:** Departamento de Biotecnología, Universidad Autónoma Metropolitana Unidad Iztapalapa, Av. San Rafael Atlixco 186, Col. Vicentina, Ciudad de México 09340, Mexico; j.j.hurtado@xanum.uam.mx (J.J.H.-R.); acym@xanum.uam.mx (Y.M.A.-C.); hbeb@xanum.uam.mx (H.E.-B.); em.carmenhdz@gmail.com (C.G.H.-V.); lpch@xanum.uam.mx (M.d.L.P.-C.); ma.pintorj@izt.uam.mx (M.A.P.-J.); jss@xanum.uam.mx (J.S.-S.); gmta@xanum.uam.mx (G.M.T.-A.)

**Keywords:** agro-industrial by-products, biorefinery, circular economy, green extraction technologies, polyphenols, natural pigments, dietary fiber and prebiotics, lipids, bioactive peptides

## Abstract

This review critically analyzes bioactive compounds derived from agro-industrial by-products, including polyphenols, natural pigments, dietary fiber, prebiotics, lipids, proteins, and bioactive peptides. The review examines their chemical characteristics, major agro-industrial sources, and recovery strategies, highlighting both conventional technologies and emerging green technologies, such as ultrasound-assisted extraction, supercritical fluids, and natural deep eutectic solvents (NADESs). Across compound classes, common patterns are identified, including the importance of external plant tissues as primary biological reservoirs, as well as a methodological convergence in extraction processes despite the wide chemical diversity of the molecules. Shared challenges related to compound stability, scalability, and process efficiency are also discussed. The results demonstrate that agro-industrial by-products should be understood as complex, integrated matrices rather than isolated sources of individual compounds, thereby supporting the development of unified biorefinery schemes. Unlike previous reviews focused on individual compound classes, this review integrates multiple classes of bioactive compounds, green extraction technologies, analytical characterization strategies, and industrial valorization approaches within a circular biorefinery framework. In conclusion, this review helps bridge the current fragmented understanding of waste valorization and highlights key opportunities for the sustainable development of high-value-added functional ingredients within the framework of the circular economy.

## 1. Introduction

The agro-food industry generates significant quantities of by-products throughout processing operations, particularly from fruits, vegetables, cereals, oilseeds, and animal-derived materials. It has been estimated that approximately 1.6 billion tons of food are lost or wasted annually, highlighting a critical global challenge associated with environmental impact, greenhouse gas emissions, and inefficient resource utilization [1,2,3]. These concerns have driven increasing interest in strategies that promote the efficient valorization of agro-industrial by-products. In this review, the term “agro-industrial by-products” is used consistently to refer to residual streams generated during food processing, in order to standardize terminology across different compound classes.

Beyond their traditional classification as waste streams in global reports, agro-industrial by-products are now recognized as complex matrices rich in recoverable bioactive compounds. These include polyphenols, natural pigments, dietary fiber, lipids, proteins, and bioactive peptides, which remain in plant and biological tissues after primary processing [4,5]. These compounds differ in chemical structure, stability, and functionality, yet they frequently coexist within the same raw material.

Bioactive compounds are naturally occurring molecules associated with a wide range of biological and technological functions. Polyphenols and pigments are widely recognized for their antioxidant and functional properties, while dietary fiber and prebiotics contribute to gastrointestinal health and metabolic regulation. Lipids, including fatty acids and phytosterols, play key roles in nutrition and food structure, whereas proteins and peptides provide both nutritional value and specific biological activities [6,7]. This diversity underscores the importance of considering these compounds not as isolated entities but as components of interconnected systems within agro-industrial matrices.

From a technological perspective, the recovery of these compounds is strongly dependent on extraction strategies adapted to the physicochemical characteristics of each compound class. Conventional solvent extraction methods remain widely used; however, increasing attention has been directed toward emerging approaches such as ultrasound-assisted extraction, supercritical fluid extraction, and natural deep eutectic solvents [8,9]. These extraction technologies play a central role in determining yield, selectivity, compound stability, and commercial applicability.

Importantly, similar extraction principles are applied across different classes of bioactive compounds despite their chemical diversity. Outer plant tissues such as peels and pomace are frequently identified as sources of polyphenols and pigments, whereas seed and bran fractions commonly contain lipids and dietary fiber [10,11]. This overlap highlights the multifunctional nature of agro-industrial by-products and supports integrated valorization approaches.

Recovered bioactive compounds have broad applications across diverse commercial sectors. In food systems, they are incorporated as functional ingredients, natural additives, and preservatives. In nutraceutical and pharmaceutical applications, they contribute to the development of health-promoting formulations, while in cosmetic products they are valued for their antioxidant and protective properties [5,7]. These applications are directly influenced by extraction efficiency, compound stability, and matrix compatibility.

Despite extensive research, most studies have addressed individual classes of bioactive compounds in isolation, leading to a fragmented understanding of agro-industrial by-product valorization [8,9,12]. This fragmentation limits the identification of shared challenges related to extraction, stability, and scalability, as well as the development of integrated processing strategies.

Accordingly, the present review provides a comprehensive and integrative analysis of bioactive compounds derived from agro-industrial by-products, including polyphenols, natural pigments, dietary fiber and prebiotics, lipids, proteins, and peptides. Unlike previous reviews focused on individual compound classes or specific residues, this review examines these compounds within a unified circular biorefinery framework, integrating their sources, extraction technologies, biological properties, analytical characterization, and end-use applications. By doing so, it provides a multidisciplinary overview of current advances, shared challenges, and future opportunities for sustainable by-product valorization [5,8,12].

**Figure 1:**
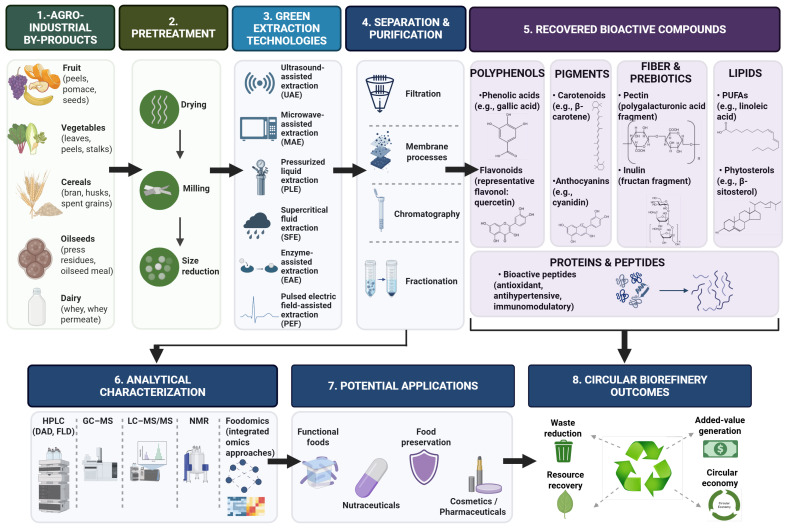
Circular biorefinery framework for agro-industrial by-product valorization.

Figure 1 summarizes the integrated valorization pathway discussed throughout this review. Agro-industrial by-products are presented as multifunctional matrices containing diverse classes of bioactive compounds that can be recovered through green extraction technologies and subsequent purification processes. The recovered bioactive fractions are characterized using advanced analytical platforms and may be incorporated into food, nutraceutical, pharmaceutical, and cosmetic applications. This holistic approach supports circular biorefinery strategies aimed at maximizing resource efficiency and generating high-value products from agro-industrial residues.

### Literature Search Strategy

This review was developed to examine the recovery, characterization, and valorization of bioactive compounds from agro-industrial by-products within a circular biorefinery framework. The document selection process was guided by the Preferred Reporting Items for Systematic Reviews and Meta-Analyses (PRISMA) statement guidelines. A total of 200 records were initially identified through the Scopus, Web of Science, PubMed, Google Scholar, ScienceDirect, SpringerLink, and Wiley Online Library databases. After consolidating the databases, 80 duplicate records were removed. The remaining 120 articles underwent an initial screening based on title and abstract, resulting in the exclusion of 12 records that did not directly align with the research objectives. Subsequently, 108 articles were evaluated in full text, and 10 were further excluded due to a lack of integration with green extraction or biorefinery frameworks. Finally, 98 articles met all eligibility criteria and were included in the final analysis.

Literature was collected using combinations of keywords related to agro-industrial by-products, bioactive compounds, polyphenols, pigments, dietary fiber, prebiotics, lipids, peptides, green extraction technologies, analytical characterization, circular economy, biorefinery, and food applications. Priority was given to peer-reviewed publications from the last five years, while seminal references were included when necessary. Articles were selected according to their relevance to bioactive compound recovery, extraction technologies, biological activities, analytical characterization, sustainability, and potential industrial applications.

## 2. Polyphenols from Agro-Industrial By-Products

### 2.1. Agro-Industrial Waste Generation and Circular Economy Context

The processing of fruits and vegetables generates substantial quantities of agro-industrial by-products at multiple stages of the food supply chain, including processing, retail, and consumption. The increasing volume of these residues has raised global concerns due to their environmental, economic, and social implications. According to the Food and Agriculture Organization (FAO), approximately 1.6 billion tonnes of food suitable for human consumption are wasted annually worldwide, with more than 80% corresponding to edible portions of food [1]. These losses highlight the urgent need for sustainable resource management and improved recovery strategies.

In response to this challenge, the circular economy has emerged as a promising model that promotes the efficient use of resources through recycling, reuse, and recovery of valuable compounds from agro-industrial by-product streams. Unlike the traditional linear production model, the CE approach aims to extend the lifecycle of materials and transform industrial residues into value-added products. The agro-food industry is particularly suitable for implementing CE strategies due to the large amounts of organic waste generated during processing activities. Within this framework, agro-industrial by-products can be valorized as sources of bioactive compounds, including polyphenols, which can be recovered and reused in various industrial applications [13,14]. This strategy reinforces the integrated biorefinery concept developed throughout this review, where agro-industrial by-products are considered key sources of multiple classes of bioactive compounds.

### 2.2. Chemical Characteristics and Biological Importance of Polyphenols

Polyphenols are a diverse group of plant secondary metabolites characterized by the presence of at least one aromatic ring bearing one or more hydroxyl groups. These compounds may occur in free form or conjugated as esters, ethers, or glycosides. Structurally, this diverse family encompasses a wide range of molecules, including phenolic acids, flavonoids, tannins, stilbenes, lignans, coumarins, and quinones. Their biosynthesis primarily occurs through the shikimate pathway, the acetate/malonate pathway, or a combination of both metabolic routes [15,16].

Polyphenols have attracted considerable attention due to their wide range of biological activities, including antioxidant, antimicrobial, anti-inflammatory, and anticancer properties. These functional characteristics have generated increasing interest in their application as active ingredients in foods, pharmaceuticals, nutraceuticals, and cosmetic products. Furthermore, global demand for these compounds has increased significantly in recent years, reflecting the increasing interest in natural bioactive compounds derived from sustainable sources [15]. These biological properties support their relevance as key target compounds in the valorization of agro-industrial by-products within circular economic strategies.

### 2.3. Occurrence and Distribution of Polyphenols in Agro-Industrial By-Products

Agro-industrial by-products constitute one of the most abundant and underutilized sources of polyphenolic compounds. During the processing of fruits, vegetables, cereals, and oil-bearing crops, substantial quantities of peels, pomaces, seeds, shells, and other residual fractions are generated. These materials often retain significant concentrations of bioactive compounds that were not recovered during primary processing. Consequently, these residual materials have gained increasing attention as sustainable feedstocks for the recovery of high-value polyphenols within circular bioeconomy and biorefinery frameworks [5].

The occurrence and concentration of polyphenols in agro-industrial residues are highly dependent on botanical origin, cultivar, geographical conditions, maturity stage, agronomic practices, and processing operations. In general, these compounds are unevenly distributed within plant tissues and tend to accumulate in external structures such as peels, skins, and seeds, where they contribute to plant defense mechanisms against environmental stressors, pathogens, and oxidative damage [6].

Among the most extensively investigated by-products, grape pomace represents a particularly rich source of phenolic compounds. Generated during wine production, grape pomace consists primarily of skins, seeds, and stems remaining after juice extraction. This by-product contains substantial concentrations of flavan-3-ols, including catechin and epicatechin, as well as flavonols such as quercetin derivatives and stilbenes such as resveratrol. Numerous studies have demonstrated that grape pomace extracts exhibit strong antioxidant activity and considerable potential for application in functional foods, nutraceuticals, and natural preservative systems [17].

Similarly, olive pomace generated during olive oil production constitutes an important source of phenolic compounds, particularly hydroxytyrosol, tyrosol, oleuropein derivatives, and other secoiridoids. These compounds are recognized for their potent antioxidant, anti-inflammatory, and cardioprotective properties. The large volume of olive pomace generated annually in Mediterranean countries has stimulated extensive research focused on the recovery of phenolic compounds as ingredients for food, cosmetic, and pharmaceutical applications [18].

Pomegranate peel is another highly valuable agro-industrial by-product characterized by exceptionally high concentrations of hydrolyzable tannins, particularly punicalagins and ellagic acid derivatives. Several investigations have reported that pomegranate peel contains substantially higher total phenolic contents than the edible arils, making it an attractive raw material for the development of natural antioxidants, antimicrobial agents, and active packaging materials. The strong antioxidant capacity of pomegranate peel extracts has been attributed primarily to the presence of ellagitannins and related phenolic constituents [19].

Citrus processing residues, including orange, lemon, grapefruit, and mandarin peels, also represent significant sources of polyphenols. These by-products are particularly rich in flavanones such as hesperidin, narirutin, naringin, and eriocitrin, which have been associated with antioxidant, anti-inflammatory, and metabolic health benefits. Given the substantial quantities of citrus waste generated worldwide, these residues offer considerable opportunities for sustainable valorization through the recovery of bioactive compounds [5].

Another important example is apple pomace, a by-product of juice and cider manufacturing, which contains appreciable amounts of chlorogenic acid, phloridzin, quercetin glycosides, and other phenolic constituents. Although the total phenolic content of apple pomace is generally lower than that of grape or pomegranate residues, its widespread availability and low cost make it an attractive source of functional ingredients. Recent studies have highlighted the potential application of apple pomace extracts as antioxidant additives in food formulations and nutraceutical products [20].

Likewise, berry processing residues, including those derived from blackberry, blueberry, raspberry, and cranberry production, are characterized by elevated concentrations of anthocyanins, flavonols, and phenolic acids. These compounds contribute not only to the antioxidant capacity of berry extracts but also to their natural coloring properties. Consequently, berry by-products have attracted increasing attention as sources of natural colorants and functional ingredients for the food industry [5,21].

The increasing recognition of agro-industrial by-products as reservoirs of polyphenolic compounds has stimulated the development of innovative extraction technologies and downstream recovery processes. However, the economic feasibility and environmental sustainability of polyphenol valorization depend not only on compound abundance but also on extraction efficiency, process scalability, solvent requirements, and integration within circular bioeconomy systems. Therefore, a comprehensive evaluation of extraction technologies is essential for the effective utilization of these resources, as discussed in the following section.

While technology-oriented comparisons provide valuable information regarding process sustainability and industrial feasibility, the practical relevance of polyphenol recovery strategies is ultimately determined by experimentally validated process performance. Representative experimental studies, including information on source materials, recovered compounds, extraction conditions, reported yields, and bioactivity assessments, are summarized in Supplementary Table S1. Due to differences in biomass characteristics, extraction conditions, solvents, and analytical methodologies, direct quantitative comparisons among studies should be interpreted cautiously.

## 3. Colorants

### 3.1. General Characteristics of Natural Pigments

Natural colorants are plant secondary metabolites widely distributed in fruits, vegetables, and other plant tissues. These compounds are responsible for the characteristic coloration of many plant-derived foods and play an important role in determining the visual quality and consumer acceptance of food products. In addition to their esthetic contribution, many natural pigments possess relevant biological activities, including antioxidant, anti-inflammatory, and antimicrobial effects, which have stimulated growing interest in applications in food science, nutrition, and health-related products [22,23].

In plants, pigments are localized in specific cellular compartments. Chlorophylls and carotenoids are mainly found in chloroplasts and chromoplasts, while anthocyanins and betalains accumulate in vacuoles. Evidence indicates that peels, pomaces, seeds, and outer tissues generated during fruit and vegetable processing contain higher pigment concentrations than edible fractions, making agro-industrial by-products attractive feedstocks for pigment recovery [24,25].

Based on their chemical structure and biosynthetic origin, plant-derived pigments can be classified into four major groups: chlorophylls, carotenoids, anthocyanins, and betalains. These compounds exhibit distinct physicochemical properties, extraction requirements, and industrial applications, underscoring the need for tailored extraction strategies for each pigment class.

### 3.2. Major Classes of Natural Pigments

#### 3.2.1. Chlorophylls

Chlorophylls are lipophilic green pigments essential for photosynthesis and are widely distributed in green plant tissues. Chemically, chlorophyll molecules consist of a tetrapyrrolic porphyrin ring coordinated with a central magnesium ion (Mg^2+^) and a hydrophobic phytol chain that anchors the molecule within lipid membranes [26].

They are mainly located in the thylakoid membranes of chloroplasts, where they participate in light-harvesting complexes responsible for converting solar energy into chemical energy during photosynthesis. Chlorophylls absorb light primarily in the blue (~430 nm) and red (~660 nm) regions of the electromagnetic spectrum while reflecting green wavelengths, giving plants their characteristic coloration [26].

Green plant residues generated during food processing, including spinach leaves, lettuce outer leaves, broccoli stems, and herb trimmings, represent promising reservoirs of chlorophylls for recovery and subsequent use as natural green colorants for food, cosmetic, and nutraceutical applications. This underscores the role of leafy vegetable residues as relevant matrices, suggesting that green biomass streams remain underexploited sources of functional pigments within agro-industrial systems [25].

#### 3.2.2. Carotenoids

Carotenoids are lipophilic pigments widely distributed in plant tissues, particularly in chloroplasts and chromoplasts. Their chemical structure consists of a long polyene chain with multiple conjugated double bonds responsible for their characteristic yellow, orange, or red coloration [27].

More than one hundred members of this pigment family have been identified in nature, including nutritionally important compounds such as β-carotene, lycopene, lutein, and zeaxanthin. In plants, carotenoids contribute to photoprotection and participate in light-harvesting processes, while in human nutrition, these compounds act as antioxidants and, in some cases, as precursors of vitamin A [23,27].

Several agro-industrial by-products represent important alternative sources for carotenoid recovery. For example, carrot peels, tomato pomace, mango skins, and citrus processing waste contain significant quantities of carotenoids that can be extracted and utilized as natural colorants or nutraceutical ingredients. Collectively, these examples demonstrate the dual role of carotenoids as both coloring agents and bioactive compounds, which indicates their relevance as multifunctional ingredients within integrated valorization strategies [25].

#### 3.2.3. Anthocyanins

Anthocyanins are water-soluble pigments belonging to the flavonoid family and are responsible for the red, purple, and blue coloration observed in many fruits, vegetables, and flowers [28]. Structurally, anthocyanins are glycosylated derivatives of anthocyanidins that share a flavylium cation core. Variations in hydroxylation, glycosylation, and acylation patterns generate a large diversity of anthocyanin molecules. The most common anthocyanidins include cyanidin, delphinidin, pelargonidin, peonidin, petunidin, and malvidin [28].

Anthocyanins are abundant in fruits such as grapes, blueberries, blackberries, cherries, and pomegranates, as well as in vegetables such as red cabbage and purple corn. In many of these plants, these pigments are concentrated in the outer tissue layers. Consequently, agro-industrial by-products such as grape pomace, berry skins, and pomegranate peels represent valuable sources of anthocyanins that can be recovered for the production of natural colorants and antioxidant-rich extracts.

#### 3.2.4. Betalains

Betalains are water-soluble nitrogen-containing pigments mainly found in plants belonging to the order Caryophyllales. These pigments produce colors ranging from yellow to red-purple and are divided into two main groups: betacyanins, which generate red to violet hues, and betaxanthins, which produce yellow to orange colors [29].

Betalains are biosynthesized from the amino acid L-tyrosine and exhibit notable antioxidant properties. Structural variations, particularly glycosylation patterns, influence their stability and functional characteristics [30].

Beetroot processing residues, including peels, pomace, and pulp generated during juice production, represent one of the most important industrial sources of betalains. These by-products can be valorized through pigment extraction to produce natural food colorants and functional ingredients. These applications illustrate the direct transition from agro-industrial residue to high-value functional ingredients, which underscores their role within circular economy frameworks [25].

### 3.3. Biological and Functional Properties

Natural pigments are not only responsible for coloration but also possess a variety of biological activities beneficial to human health. Chlorophylls and their derivatives have been reported to exhibit antioxidant properties by scavenging reactive oxygen species and protecting cellular components from oxidative damage [31].

Carotenoids also function as potent antioxidants and have been associated with protective effects against several chronic diseases, including cardiovascular disorders and metabolic diseases [23].

Similarly, anthocyanins display strong antioxidant and anti-inflammatory activities and have been linked to cardioprotective, neuroprotective, and antidiabetic effects. These bioactivities further support the relevance of pigment recovery from agro-industrial by-products as a strategy for developing functional food ingredients [32]. In addition, the antioxidant capacity of natural pigments contributes to improving oxidative stability in food systems, supporting their application as multifunctional ingredients that provide both technological and health-related benefits [23,31,32]. Betalains have also attracted attention due to their antioxidant and anti-inflammatory properties, expanding the range of bioactive pigments that can be recovered from agro-industrial residues for functional applications [24]. Collectively, these properties reinforce their role in the development of health-promoting systems, in line with other bioactive compounds discussed in this review.

## 4. Dietary Fiber and Prebiotics

### 4.1. General Characteristics of Dietary Fiber

Dietary fiber is defined as remnants of edible plant cells that are resistant to digestion by human enzymes. It is generally classified into soluble and insoluble fractions according to its solubility in water. Soluble fiber mainly includes pectin, gums, and mucilage, while insoluble fiber consists primarily of cellulose, hemicellulose, and lignin. Although fiber is not considered an essential nutrient, it plays a fundamental role in maintaining intestinal health and physiological homeostasis [33,34]. These compositional characteristics are particularly relevant when considering agro-industrial by-products, where fiber composition directly influences both technological functionality and potential applications.

The American Association of Cereal Chemists (AACC) later proposed a widely accepted definition, describing dietary fiber as the edible parts of plants or analogous carbohydrates that are resistant to digestion and absorption in the small intestine and undergo complete or partial fermentation in the large intestine. These evolving definitions reflect the increasing recognition of dietary fiber as a functional component with important physiological roles [33].

The recommended daily intake of dietary fiber ranges from 25 to 35 g. Among its physiological effects are reduced intestinal transit time, increased stool volume, decreased postprandial blood glucose levels, and reduced cholesterol concentrations. Fiber also promotes satiety and lowers the glycemic index by delaying nutrient absorption and increasing the viscosity of intestinal contents [34]. These physiological effects explain the growing interest in recovering dietary fiber from agro-industrial by-products as functional food ingredients.

### 4.2. Prebiotics and Their Physiological Effects

Some dietary fibers are resistant to enzymatic digestion in the small intestine but are susceptible to fermentation by microorganisms in the colon. During this fermentation process, gut microbiota produce short-chain fatty acids (SCFAs), mainly acetate, propionate, and butyrate, which contribute to intestinal health and metabolic regulation [35,36].

Prebiotics are defined as non-digestible ingredients that confer health benefits to the host by selectively stimulating the growth or activity of beneficial intestinal microorganisms [35]. More recently, prebiotics have been defined as substrates that are selectively utilized by host microorganisms, resulting in health benefits [36].

To be classified as a prebiotic, a compound must meet several criteria, including resistance to digestion in the upper gastrointestinal tract, fermentability by intestinal microbiota, and selective stimulation of beneficial bacterial populations [37]. Fructan-type inulin is considered the model prebiotic compound because it fulfills these criteria. Other substrates with prebiotic potential include fructooligosaccharides, galactooligosaccharides, β-glucans, arabinoxylans, mannan oligosaccharides, lactulose, pectin, and xylooligosaccharides [38]. Many of these compounds can be derived from agro-industrial by-products, reinforcing their relevance as sustainable sources of functional ingredients.

Accumulating evidence indicates beneficial effects of prebiotics in the prevention or management of several health conditions, including colorectal cancer, metabolic syndrome, obesity, diabetes, and intestinal disorders such as irritable bowel syndrome. These health effects support incorporating prebiotic compounds into functional food formulations to improve host health [39]. This functional relevance aligns with the biological activities described for other classes of bioactive compounds in this review, such as polyphenols and pigments.

### 4.3. Agro-Industrial By-Products as Sources of Fiber and Prebiotics

Agro-industrial by-products derived from fruit and vegetable processing are valuable sources of dietary fiber and potential prebiotics. In many cases, non-edible plant tissues such as peels, seeds, and pomace contain high concentrations of structural carbohydrates, including cellulose, hemicellulose, pectin, and lignin [40,41].

The fiber composition of plant residues varies with plant species, tissue type, and stage of ripening. For instance, the cellulose and lignin content often increases during fruit maturation because these compounds provide structural stability to plant tissues [41,42].

Various investigations have evaluated the fiber content of agro-industrial by-products. The fiber content of cactus pear and pineapple peels has been evaluated, reporting values of 64.15% and 62.54%, respectively, with a soluble-to-insoluble fiber ratio of approximately 50% in cactus pear peel and 35/65 in pineapple peel [40].

Similarly, a dietary fiber content of 70.91% has been reported in apple pomace generated during cider production, with the majority corresponding to insoluble fiber [42].

Other agro-industrial by-products have also been evaluated as sources of dietary fiber. Several by-products, including lemon peel, grapefruit peel, pomegranate peel, lemon albedo, and tiger nut residues, have shown fiber contents ranging from 40 to 70%, with a predominance of insoluble fiber fractions in most samples [41]. These observations reinforce the relevance of agro-industrial by-products as fiber-rich materials for functional food applications, which indicates their potential to contribute to both nutritional enhancement and waste reduction strategies [40,41]. Overall, these materials represent concentrated sources of bioactive compounds, as discussed in previous sections.

The prebiotic potential of fibers derived from agro-industrial by-products has been evaluated through both in vivo and in vitro studies. The physiological effects of cactus pear peel flour and apple pomace flour have been evaluated in Wistar rats, using inulin as a control prebiotic, showing that animals fed apple pomace exhibited weight gains similar to those observed with inulin, while cactus pear peel promoted higher counts of lactic acid bacteria and bifidobacteria, suggesting potential prebiotic activity [43].

Another approach to evaluating prebiotic potential is calculating the prebiotic activity index, which measures the selective stimulation of probiotic bacteria by specific substrates. Prebiotic activity values of 1.17 for *Lactobacillus paracasei* using inulin as a substrate have been reported, whereas negative values were obtained for *Bifidobacterium bifidum* with galactooligosaccharides, highlighting the selective nature of prebiotic substrates [44].

Prebiotic activity values for agro-industrial by-products are often lower than those observed for purified prebiotics. This is mainly because these residues typically contain higher proportions of insoluble fiber, which is more difficult for microorganisms to degrade. Consequently, microbial growth may occur in two phases (diauxic growth), during which microorganisms gradually adapt to the new substrate. This behavior emphasizes the influence of substrate composition on microbial adaptation and prebiotic efficiency, suggesting that structural complexity is a key factor influencing the functional performance of agro-industrial fibers [44].

A summary of the main agro-industrial by-products used as sources of dietary fiber and the prebiotic index is presented in Table 1.

## 5. Lipids—Fatty Acids and Triacylglycerols, Phospholipids, Sterols and Minor Lipids, and Fat-Soluble Vitamins

### 5.1. Characteristics

Lipids constitute a diverse group of hydrophobic or amphiphilic biomolecules that perform essential structural, energetic, and regulatory roles in biological systems [45,46]. In food matrices, lipids strongly influence texture, flavor release, mouthfeel, oxidative stability, and nutritional quality. They also function as carriers of fat-soluble vitamins (A, D, E, and K) and other lipophilic bioactive compounds [47,48].

Chemically, lipids encompass several classes including fatty acids, triacylglycerols, phospholipids, sterols, and minor lipid components such as tocopherols and squalene [46,47]. During food processing, particularly in the industrial processing of oilseeds, fruits, cereals, and nuts, large quantities of lipid-rich by-products are generated, including seeds, pomace, bran fractions, and deodorizer distillates [46,48].

**Figure 2:**
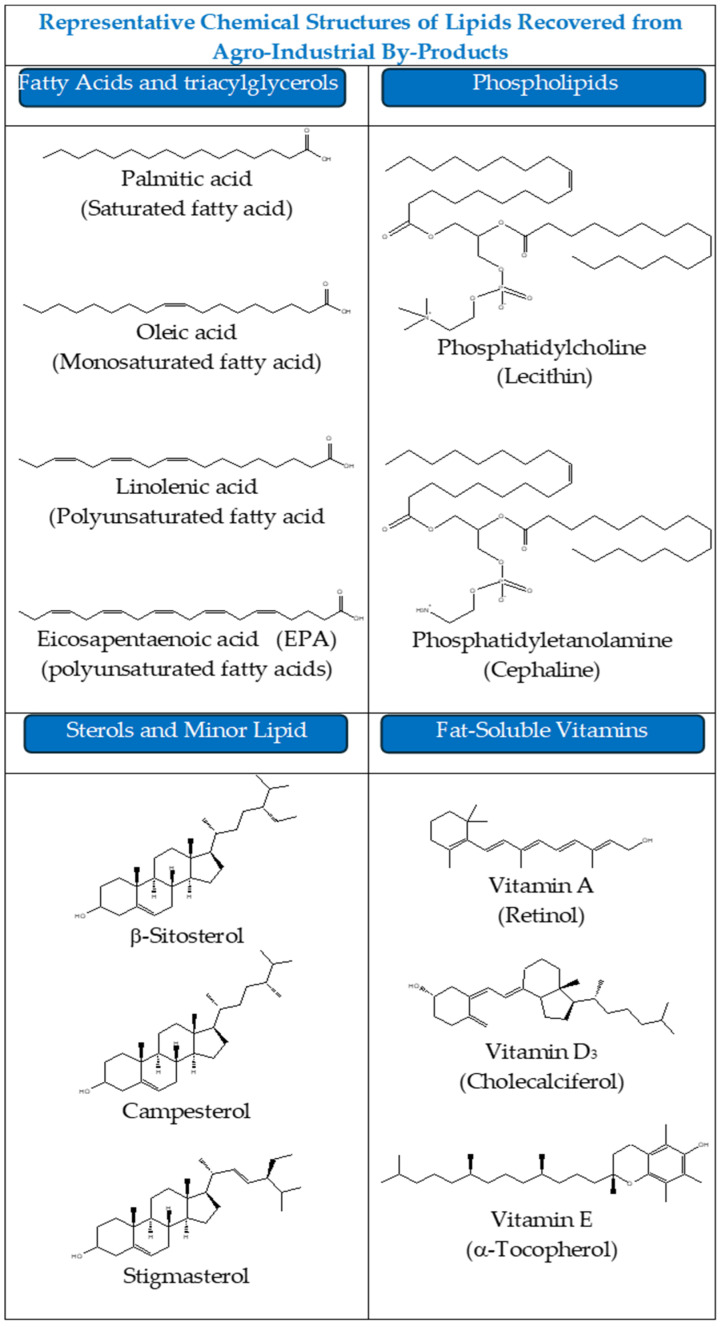
Representative chemical structures of lipid classes recovered from agro-industrial by-products.

The structural diversity of lipid fractions recovered from agro-industrial by-products is illustrated in Figure 2. Major lipid classes include fatty acids, triacylglycerols, phospholipids, phytosterols, and fat-soluble vitamins, each contributing distinct nutritional, technological, and biological functions. Collectively, these lipid classes underlie the broad range of applications of recovered lipid fractions in food, nutraceutical, cosmetic, and pharmaceutical formulations [46,47,48,49].

These secondary streams represent valuable reservoirs of recoverable lipid fractions that can be transformed into edible oils, phytosterol concentrates, lecithins, and structured lipids. The recovery and utilization of these compounds support circular bioeconomy strategies and contribute to the sustainable valorization of agro-industrial by-products [46,49]. A comparable pattern is observed across other classes of bioactive compounds discussed throughout this review, which indicates that lipid fractions should be considered integral components of multi-compound valorization strategies rather than isolated resources.

### 5.2. Fatty Acids and Triacylglycerols

Fatty acids are classified according to their degree of unsaturation as saturated, monounsaturated, or polyunsaturated fatty acids [45]. Triacylglycerols represent the primary storage lipids in plant tissues and constitute the dominant fraction of most edible oils [48].

Several agro-industrial by-products contain lipid fractions rich in nutritionally relevant fatty acids. For example, grape seeds, mango kernels, rice bran, and olive pomace contain significant levels of oleic, linoleic, and α-linolenic acids [45,46]. The industrial recovery of these lipid fractions enables the production of cold-pressed oils, nutraceutical oils enriched in omega-3 fatty acids, and structured lipids used in functional food formulations [50].

Rice bran oil obtained from milling by-products represents a particularly valuable example, as it contains γ-oryzanol and other antioxidant compounds that contribute to lipid metabolism regulation and oxidative stability [47]. Similarly, grape-seed oil derived from winery residues has been widely valorized for culinary, nutraceutical, and cosmetic applications [48]. Together, these examples demonstrate the potential of agro-industrial by-products as sources of nutritional and technologically valuable lipid fractions, supporting their role within biorefinery strategies.

### 5.3. Phospholipids

Phospholipids are amphiphilic molecules that form the structural basis of biological membranes and possess natural emulsifying properties [45]. In industrial processes, phospholipids are mainly recovered from lecithin streams generated during vegetable-oil refining [48]. These fractions can be purified to obtain standardized lecithins that are widely used as natural emulsifiers in bakery products, confectionery formulations, and dairy systems. Moreover, phospholipids are increasingly employed in advanced delivery systems such as liposomes and nano-emulsions designed to enhance the bioavailability of lipophilic bioactive compounds [46].

The recovery of phospholipids from oil-refining streams demonstrates how agro-industrial by-products can be converted into high-value functional ingredients with applications in food, pharmaceutical, and cosmetic sectors. This strategy supports circular bioeconomy principles focused on the valorization of agro-industrial by-products across multiple compound classes [49]. Similarly, phospholipid recovery follows a valorization pattern comparable to that described for other bioactive compounds discussed in this review, supporting a unified approach to by-product utilization.

### 5.4. Sterols and Minor Lipids

Minor lipid components such as phytosterols, tocopherols, and squalene possess significant biological and technological value [45,47]. These compounds are frequently recovered from vegetable-oil deodorizer distillates using molecular distillation and fractionation techniques [46,48].

Phytosterols are commonly incorporated into functional foods due to their ability to reduce serum cholesterol levels by inhibiting intestinal cholesterol absorption [47]. Tocopherols act as natural antioxidants that improve lipid stability, while squalene is widely used in cosmetic formulations because of its antioxidant and skin-protective properties [45].

A representative example of agro-industrial valorization is the recovery of squalene from olive pomace, which is currently utilized in nutraceutical products and cosmetic formulations due to its emollient and antioxidant properties. Such examples illustrate the high added value that can be obtained from lipid-rich residues within integrated biorefinery schemes [49].

### 5.5. Fat-Soluble Vitamins

Fat-soluble vitamins constitute an important group of lipophilic micronutrients commonly associated with lipid fractions recovered from agro-industrial by-products. These include vitamins A, D, E, and K, which are involved in key physiological processes such as antioxidant protection, immune regulation, and cellular metabolism [45,47].

Vitamin E compounds, including tocopherols and tocotrienols, are among the most frequently recovered vitamins from agro-industrial by-products. Oilseed by-products such as rice bran, grape seeds, sunflower seeds, and wheat germ contain considerable amounts of these compounds, which contribute to the oxidative stability of oils and exhibit antioxidant properties [45,48].

Furthermore, carotenoid-rich oils obtained from fruit residues such as mango kernels and tomato seeds may act as precursors of provitamin A compounds [49]. Leafy vegetable residues generated during processing may also contain vitamin K derivatives associated with chloroplast membranes, which can be co-extracted during lipid recovery processes [46].

The recovery of fat-soluble vitamins from agro-industrial by-products contributes to improving the nutritional value of recovered lipid fractions while enabling the development of functional ingredients and nutraceutical formulations within circular bioeconomy frameworks. These observations reinforce the relevance of lipid fractions as multifunctional carriers of bioactive compounds in sustainable food systems [46,49].

### 5.6. Biological Activity of Lipids Derived from Agri-Industrial By-Products

Beyond their role as energy reserves and structural components, lipid fractions recovered from agro-industrial by-products exhibit diverse biological activities that contribute to their nutritional and functional value [45,47]. These bioactivities are largely associated with the presence of unsaturated fatty acids, phytosterols, phospholipids, tocopherols, and other minor lipophilic constituents naturally retained in plant-derived residues.

Polyunsaturated fatty acids recovered from oilseeds, fruit pomaces, and fish-processing residues have been associated with antioxidant, anti-inflammatory, and cardioprotective effects. In particular, omega-3 fatty acids may contribute to the modulation of inflammatory responses and oxidative stress, while unsaturated fatty acids can influence lipid metabolism and cardiovascular health [45,48].

Phytosterol-rich fractions exhibit hypocholesterolemic activity by reducing intestinal cholesterol absorption, whereas tocopherols and other vitamin E derivatives act as natural antioxidants capable of protecting lipids and cellular components against oxidative damage [45,47]. In addition, phospholipids contribute to membrane stability and participate in physiological processes related to cellular signaling and nutrient transport [46,48].

The coexistence of these bioactive lipid constituents within agro-industrial residues highlights the biological potential of recovered lipid fractions. Together, these bioactivities support continued interest in lipid recovery as part of sustainable agro-industrial valorization strategies. A summary of representative lipid fractions, major bioactive compounds, reported biological activities, and associated applications is presented in Table 2 [46,47,48,49].

## 6. Proteins and Peptides

### 6.1. General Characteristics and Recovery from By-Products

Bioactive peptides are short sequences of amino acids, typically composed of 2–20 residues with molecular weights ranging from approximately 0.4 to 2 kDa, which are released from parent proteins through enzymatic hydrolysis, microbial fermentation, or gastrointestinal digestion [51,52]. While these peptides are encrypted within the primary structure of proteins and remain inactive in the native state, proteolytic processes can liberate them, allowing them to exert diverse biological activities beneficial to human health.

Food processing industries generate large quantities of protein-rich residues that can serve as valuable substrates to produce bioactive peptides. Such residues include dairy whey, meat processing by-products, fish skins and bones, poultry residues, egg processing wastes, cereal bran, and oilseed cakes, which contain significant quantities of recoverable proteins [53,54]. Recovering proteins from these materials contributes to the development of sustainable food systems by transforming low-value biomass into functional compounds with nutritional and technological value.

Representative examples include the potential of these resources. Fish skins, bones, and viscera generated during seafood processing are rich in collagen and structural proteins that can be hydrolyzed to produce antioxidants or antihypertensive peptides [55]. Likewise, poultry processing residues and egg by-products have been investigated as sources of peptides exhibiting antimicrobial, antihypertensive, and enzyme-inhibitory properties [54,56]. Plant-derived residues such as oilseed cakes and fruit seeds have also been explored as sources of antioxidant and angiotensin-converting enzyme (ACE) inhibitory peptides [57].

The recovery of proteins and peptides from agro-industrial by-products therefore represents an important strategy for reducing food waste while generating high-value functional ingredients. Accordingly, protein-rich by-products can be considered key resources within circular bioeconomy frameworks, consistent with the valorization approaches described for other bioactive compounds throughout this review [53,58].

### 6.2. Biological Activities of Proteins and Peptides Derived from Agro-Industrial By-Products

Bioactive peptides derived from agro-industrial by-products exhibit a wide range of biological activities including antioxidant, antimicrobial, antihypertensive, opioid-like, and enzyme-inhibitory effects, which support their incorporation into functional foods, nutraceuticals, and pharmaceutical formulations [53,58]. These biological activities are comparable to those described for other bioactive compounds such as polyphenols, lipids, and pigments, further emphasizing the multifunctional potential of agro-industrial by-products [52].

#### 6.2.1. Antioxidant Activity

Bioactive peptides derived from food by-products may act as natural antioxidants by scavenging free radicals, chelating pro-oxidant metal ions, and inhibiting lipid peroxidation [58]. Most peptides exhibiting antioxidant activity include 4–16 amino acid residues and have a molecular mass of 0.4–2 kDa [58].

The antioxidant activity of peptides is closely related to the presence of amino acids such as histidine, proline, tyrosine, glutamic acid, phenylalanine, cysteine, tryptophan, and methionine, which facilitate electron donation and radical scavenging mechanisms [59]. Tyrosine-containing peptides primarily function through hydrogen atom transfer, whereas cysteine, tryptophan, and histidine-containing peptides primarily operate through single-electron transfer. Tyrosine, phenylalanine, and tryptophan, aromatic amino acids, can act as radical eliminators because they are excellent at donating protons to electron-deficient radicals [56,58,59]. This characteristic enhances the ability of bioactive peptides to scavenge radicals. Antioxidant peptides with leucine in their *N*-terminal carbon, such as LNQWLPHSGY and LLGPGLTNHA, can improve the capacity to capture electrons in the peptide interaction, while other dipeptides, mostly identified as histidine, carnosine, and anserine, have been highlighted because of their demonstrated antioxidant activity [59,60]. Additionally, peptides containing hydrophobic amino acids may interact more efficiently with lipid substrates, enhancing their ability to prevent oxidative deterioration in food systems [52]. Representative antioxidant peptides have been isolated from Watermelon seed [57], Buffalo horn, Duck skin [59], Chicken feathers [61], Tuna backbone, Bluefin leatherjacket heads, Squid head [62], Salted duck egg white [63] and Eggshell membrane [64].

#### 6.2.2. Antimicrobial Activity

Antimicrobial peptides (AMPs) represent an important class of bioactive compounds capable of inhibiting the growth of pathogenic microorganisms, fungi, and parasites [53]. These peptides are generally classified into three groups according to their antimicrobial properties: small (20–46 amino acid residues), basic (enriched in lysine or arginine), and amphipathic with an abundance of hydrophobic residues (leucine, isoleucine, valine, phenylalanine, and tryptophan) positively charged, which facilitate their interaction with negatively charged microbial membranes [57,58].

The antimicrobial mechanisms vary depending on the antimicrobial peptide, primarily including the barrel-stave mechanism, the toroid pore or wormhole mechanism, and the carpet mechanism with intracellular processes such as inhibition of DNA replication or protein synthesis [53]. Peptides derived from fish processing residues, poultry by-products, and dairy hydrolysates have demonstrated antimicrobial activity [53,58]. AMPs from agro-industrial by-products are generated through the hydrolysis of proteins present in these by-products; however, only a few studies have been reported to date. Representative bioactive peptides have been identified from bovine blood (STVLTSKYR) [59], anchovy cooking wastewater (GLSRLFTALK) [58], and whey protein, including lactoferricin [58]. These peptides have antimicrobial activity against *Micrococcus luteus* A270, *Listeria innocua*, *Escherichia coli*, *Salmonella enteritidis*, *Staphylococcus aureus*, *Bacillus subtilis*, *Shigella dysenteriae*, *Pseudomonas aeruginosa*, *Salmonella typhimurium*, and *Streptococcus pneumoniae* [58,59]. These findings further demonstrate the potential of agro-industrial by-products as sustainable sources of naturally occurring antimicrobial peptides for food, nutraceutical, and pharmaceutical applications.

#### 6.2.3. Antihypertensive Activity

Antihypertensive peptides derived from agro-industrial by-products exert their biological activity primarily through inhibition of the angiotensin-converting enzyme (ACE), a key regulator of blood pressure. Hypertension is one of the leading risk factors for cardiovascular disease worldwide. Several food-derived peptides exert antihypertensive effects through inhibition of ACE, which converts angiotensin I into the vasoconstrictor angiotensin II [56]. Aromatic amino acid residues at the *C*-terminus (PFY) and hydrophobic amino acid residues at the *N*-terminus (Val or Ile) help peptides block ACE function [56]. Hydrophobic amino acids with low molecular weights, such as Pro or Ala, can interact hydrophobically in the active site of ACE, inhibiting its activity and acting as an antihypertensive agent and regulator of blood pressure [58]. Some inhibitory peptides that have a positive effect on reducing blood pressure in animal models and humans are LYSPH, LYTPH, HLLP, VPP, and IPP [60].

These peptides have been identified in hydrolysates derived from Whey protein hydrolysate [52], Bovine blood [59], Chicken skin, Poultry viscera, Chicken legs [56], Palm kernel oil cake [57], Tilapia skin and Olive flounder surimi [62], highlighting their potential application in functional foods designed to support cardiovascular health [53].

#### 6.2.4. Opioid Activity

Food-derived opioid peptides frequently contain characteristic N-terminal sequences such as YGGF, YLGYLE, TLGYL, RYLGYLE, GYYPTGYYPYGGWLYGGW, and YPISL that are essential for receptor recognition. Such peptides can be generated during enzymatic hydrolysis of proteins from milk, dairy products, and milk by-products, as well as in various plant sources, and they have been investigated for their potential roles in appetite regulation, stress response modulation, and gastrointestinal function [58,59,64,65]. Additionally, these biological compounds are characterized as small molecules of 5–80 amino acids that actively interact with opioid receptors in the nervous and gastrointestinal systems, producing crucial effects such as analgesia, neuromodulation, and regulation of gastrointestinal motility [66].

#### 6.2.5. Prolyl Endopeptidase Inhibitory Activity

Prolyl endopeptidase (PEP)-inhibitory peptides represent an emerging class of bioactive peptides with potential applications in cognitive health. Dysregulation of PEP has been associated with neurological disorders, including Alzheimer’s disease and Parkinson’s disease, and may also contribute to alterations in learning, memory, mood, eating behavior, and other central nervous system functions [67,68].

Several peptides derived from food by-product proteins have demonstrated potential as natural PEP inhibitors, which improve memory, learning disorders and neurological health [53,67]. Representative PEP inhibitory peptide sequences include MPPPLPARVDFSLAGALN, derived from bovine glial fibrillary acidic protein in bovine brain; PPPPGGPQPRPPQG, derived from bovine albumin and collagen; and LVVYPWTQRF from bovine blood [64,67,68].

#### 6.2.6. DPP-IV Inhibitory Activity

DPP-IV inhibitory peptides have attracted increasing interest because of their potential role in the management of type 2 diabetes mellitus. These peptides exert their biological activity by inhibiting dipeptidyl peptidase-IV (DPP-IV), an enzyme involved in glucose metabolism through the degradation of incretin hormones responsible for stimulating insulin secretion [69].

Several peptides derived from egg processing residues (Eggshell membrane), poultry by-products (Chicken claws), and marine (Shrimp head) processing waste have demonstrated promising DPP-IV inhibitory activity and are currently being investigated as potential nutraceutical compounds for glycemic control [52,67,69].

## 7. Advanced Sensory Evaluation Methodologies for Food Products Enriched with Bioactive Compounds

### 7.1. Foods Developed from Agro-Industrial By-Products

The agro-food industry faces the challenge of managing the excessive generation of by-products resulting from food production in a sustainable manner. This increase is associated with rapid urban development, population growth, and the continuous demand for food products with higher added value. In this context, the reuse of agro-industrial by-products has emerged as an important strategy to reduce environmental impact, promote circular economy practices, and encourage the development of innovative food products [70]. These by-products represent valuable sources of bioactive compounds, proteins, and dietary fiber that may contribute to improving the nutritional quality of food formulations. However, beyond their nutritional value, the successful incorporation of agro-industrial by-products into food products also depends on consumer perception and acceptance, making sensory evaluation a key tool in the development of these sustainable food systems [66,70,71]. This perspective complements the broader framework developed throughout this review, in which the recovery of bioactive compounds is accompanied by sensory validation to facilitate successful food applications.

The commercial success of foods containing agro-industrial by-products therefore depends not only on their nutritional and functional properties but also on their safety and consumer acceptance. Sensory evaluation plays a fundamental role in the development of these products because it allows the characterization of sensory attributes, the optimization of formulations, and the prediction of consumer responses to foods developed with agro-industrial by-products. Through analytical, hedonic, and dynamic sensory methods, it is possible to evaluate attributes such as aroma, texture, flavor, and overall acceptability, which ultimately determine the market success of newly developed foods [66,70].

Representative studies evaluating the sensory performance of foods enriched with agro-industrial by-products are summarized in Table 3, allowing comparison of incorporation levels, sensory methodologies, and principal outcomes across different food matrices [70,72]. Overall, Table 3 illustrates how the sensory impact of agro-industrial by-products depends on both the type of food matrix and the level of incorporation, illustrating consistent trends across different product categories.

### 7.2. Functional and Fortified Foods

Functional foods provide health benefits beyond basic nutrition due to the presence of bioactive compounds such as antioxidants, dietary fiber, or probiotics, whereas fortified foods contain essential nutrients including vitamins, minerals, or proteins [70]. The incorporation of agro-industrial by-products into these products provides a viable pathway to enhance their nutritional value while providing innovative alternatives for consumers [70].

Wine production generates considerable amounts of grape pomace, a by-product rich in dietary fiber and polyphenols that can be valorized as a functional ingredient in food formulations. Tomato purées enriched with grape pomace and grape skins of different particle sizes were evaluated through descriptive sensory analysis to determine their organoleptic properties, including texture (crunchiness and granularity), aroma (spicy and hay), homogeneity, and astringency [72]. Consumer acceptance was subsequently evaluated.

Larger grape pomace particles were associated with sensory attributes such as crunchiness, granularity, and vegetal or spicy aromas. Regarding consumer preference, two distinct groups were identified. One group preferred formulation containing smaller particles (<125 µm), which produced a more homogeneous texture and a flavor profile closer to conventional tomato purées. The second group showed greater preference for formulations containing larger particles (>125 µm), which were associated with attributes such as tomato freshness, crunchy texture, and vegetal notes [72].

The sensory potential of beverages formulated with agro-industrial by-products has also been investigated. Beverages produced with fermented-extruded wheat were evaluated at three incorporation levels (5, 10, and 15 g per serving), while fruit processing by-products including blueberry, raspberry, sea buckthorn, and currants were incorporated at three concentrations (2.5, 5.0, and 7.5 g per serving) [83]. Overall acceptability was evaluated by 50 consumers using a ten-point hedonic scale ranging from 0 (extremely unpleasant) to 10 (extremely pleasant).

In addition to consumer acceptance, emotional responses associated with beverage consumption were analyzed through facial expression recognition approximately 15 min after intake. Nine basic emotions were evaluated, including neutral, happy, sad, angry, surprised, scared, disgusted, and contempt, and emotional valence was also calculated. The beverage containing fermented and extruded wheat obtained the highest acceptability scores. Interestingly, the emotion “scared” showed a moderate positive correlation with product acceptability (r = 0.5295), although this response was interpreted as a positive emotional reaction. Furthermore, beverages containing fruit by-products achieved high acceptance scores, reaching values of up to 9.5 on the ten-point scale. Facial expression analysis revealed a strong positive correlation between the emotion “happy” and product acceptance (r = 0.8525), whereas negative correlations were observed between acceptance and emotions such as anger (r = −0.6842) [73].

The incorporation of agro-industrial by-products into beverage formulations can generate favorable sensory responses when the level of addition is carefully optimized. In comparative terms, beverages appear to tolerate moderate enrichment levels more readily than more texture-sensitive matrices, although consumer acceptance still depends on balancing nutritional enhancement with flavor familiarity and emotional response [70,73]. Overall, these observations indicate that sensory response is closely linked to formulation strategy, a pattern also observed in other matrices discussed in this section.

### 7.3. Snacks and Cookies

Snack foods and baked products represent suitable matrices for the incorporation of agro-industrial by-products due to their formulation flexibility and wide consumer acceptance. Several studies have therefore evaluated the sensory impact of incorporating plant processing residues into snack formulations [74].

Extruded snacks formulated with rice semolina, wheat flour, defatted hazelnut flour (5–15%), and fruit residues (3–7%) were evaluated using a nine-point hedonic scale to assess color, flavor, texture, and overall acceptability [74]. The incorporation of defatted hazelnut flour significantly influenced color (*p* < 0.05), producing darker products. However, lower levels of hazelnut flour improved crunchiness and overall acceptance.

The incorporation of Hibiscus sabdariffa processing residues into cookies has also been investigated. Sensory evaluation performed by a trained panel assessed attributes including color, aroma, flavor, crunchiness, appearance, and overall acceptance using a seven-point hedonic scale [75]. Formulations containing 1.25% and 2.50% of the by-product achieved favorable sensory scores, whereas cookies containing 5% of the residue exhibited lower acceptability.

Similarly, apple pomace has been incorporated into cookies as a functional ingredient. Partial substitution of wheat flour with 10% and 20% apple pomace was evaluated through descriptive sensory analysis including fruity flavor, baked flavor, texture, sweetness, and acidity [76]. The incorporation of apple pomace increased fruity flavor intensity without negatively affecting other sensory characteristics such as firmness, sweetness, or crunchiness.

Overall, these studies indicate that snack and bakery matrices can successfully incorporate agro-industrial by-products at moderate levels; however, excessive substitution tends to intensify color, flavor, or textural deviations that negatively affect acceptance. This pattern highlights an important comparative trend across matrices: the technological feasibility of valorization does not always translate into equivalent sensory tolerance, making formulation optimization essential [70,74,75,76]. This trend is consistently observed throughout the present review, emphasizing that improvements in functionality must be balanced with sensory acceptability.

### 7.4. Pasta and Bakery Products

Cereal-based foods such as pasta and bakery products have also been explored as suitable matrices for the incorporation of agro-industrial by-products. In these products, sensory perception is strongly influenced by both the type of ingredient incorporated and the level of substitution applied [77].

Pasta enriched with polyphenol-rich grape pomace has been evaluated to determine the effect of this by-product on product sensory quality [77]. The addition of grape pomace slightly modified pasta color, producing a light brown tone compared with the bright yellow color observed in conventional pasta. Nevertheless, sensory attributes such as flavor and texture were not significantly affected.

Another representative example involves pasta formulations enriched with fresh and dried potato juice, which were evaluated through sensory analysis and quantitative descriptive analysis [78]. Pasta containing fresh potato juice obtained acceptance scores comparable to traditional pasta (6.8 on a nine-point scale). Multivariate analysis indicated that color was the attribute with the greatest influence on consumer perception.

Additional agro-industrial by-products have been incorporated into pasta formulations. Coconut processing residues, including coconut flour (10–15%) and coconut milk by-products (5–10%), were incorporated without significantly affecting aroma, taste, or texture [79]. However, their incorporation resulted in noticeable changes in color.

In bakery products, muffins produced with partial replacement of wheat flour by cauliflower by-product flour at substitution levels of 10%, 20%, and 30% were evaluated using a nine-point hedonic scale by trained and untrained panelists [80]. Moderate substitution levels maintained acceptable sensory quality, whereas higher substitution levels negatively affected appearance and overall acceptability.

Taken together, these findings indicate that cereal-based foods can tolerate moderate incorporation levels of agro-industrial by-products, although visible color changes frequently emerge as the first limiting sensory attribute. Compared with beverages and some snack products, pasta and bakery matrices often preserve flavor acceptability more effectively, but appearance and texture remain critical determinants of consumer response [77,78,80]. Taken together, these observations confirm that visual attributes such as color often represent the first limiting factor in consumer acceptance of enriched products.

### 7.5. Meat and Dairy Products

The incorporation of agro-industrial by-products has also been investigated in animal-derived foods such as dairy products and meat formulations, where these ingredients may contribute to improved nutritional value while maintaining acceptable organoleptic properties [70].

Cheeses produced from goats fed diets supplemented with orange pulp were evaluated through sensory analysis performed by expert panelists assessing attributes including milk aroma, butter notes, sweet cake-like odors, toffee, nuts, and characteristic goat flavor [82]. Most formulations showed similar sensory profiles dominated by buttery and acidic notes.

Beef burgers enriched with flaxseed and flaxseed by-products at incorporation levels of 2.5% and 5.0% were evaluated using a nine-point hedonic scale assessing flavor, aroma, appearance, texture, and overall acceptance [81]. Moderate incorporation levels maintained acceptable sensory scores, whereas higher concentrations negatively affected flavor and texture.

Agro-industrial by-products can also be successfully incorporated into animal-derived foods without drastically compromising sensory quality. However, optimization of formulation levels remains essential to ensure consumer acceptance and product quality [70,81,82]. This underscores the importance of formulation design in maintaining sensory balance while improving nutritional value. These observations parallel those reported for plant-based matrices, reinforcing the need for product-specific optimization strategies. Overall, the reuse of agro-industrial by-products enables the development of value-added food systems that improve the nutritional profile of foods while contributing to waste reduction and circular economy goals.

Nevertheless, the sensory evidence reviewed here indicates that successful valorization depends on matrix-specific optimization, as the same by-product may improve nutritional quality while simultaneously modifying color, texture, aroma, or flavor to different extents across food categories. This comparative perspective reinforces the integrative role of sensory evaluation within the broader recovery, characterization, and application framework developed throughout this review. Overall, this integrative perspective confirms that the successful implementation of agro-industrial by-product valorization requires not only technological and biochemical optimization but also alignment with sensory performance and consumer acceptance [70,72,80].

## 8. Analytical Tools for the Characterization of Bioactive Compounds Recovered from Agro-Industrial By-Products

The valorization of agro-industrial by-products through green extraction technologies requires robust analytical strategies capable of confirming compound identity, purity, stability, and biological relevance. Although extraction yield is frequently used as a performance indicator, it does not provide sufficient information regarding the chemical composition or functional potential of recovered bioactive fractions. Therefore, analytical characterization constitutes an essential component of biorefinery systems, enabling the identification of bioactive compounds and supporting their subsequent application in food, nutraceutical, pharmaceutical, and cosmetic products [66,71].

The increasing implementation of ultrasound-assisted extraction (UAE), microwave-assisted extraction (MAE), supercritical fluid extraction (SFE), pressurized liquid extraction (PLE), and NADESs has intensified the need for analytical platforms capable of evaluating extraction selectivity and compound integrity [27,66,71,83]. Consequently, the selection of analytical tools should be guided primarily by the type of bioactive compound being recovered rather than by methodological preference alone.

### 8.1. Compound-Specific Analytical Platforms

#### 8.1.1. Polyphenols and Natural Pigments

Polyphenols and natural pigments are among the most extensively studied bioactive compounds recovered from agro-industrial by-products due to their antioxidant, antimicrobial, and anti-inflammatory properties. Initial screening is commonly performed using spectrophotometric methods such as the Folin–Ciocalteu assay and Ultraviolet–visible spectroscopy (UV-Vis) because of their simplicity and rapid implementation [16]. However, these approaches provide only global estimations and cannot differentiate individual compounds.

For detailed characterization, chromatographic techniques remain essential. High-performance liquid chromatography with diode array detection (HPLC-DAD) is widely employed for quantification of phenolic acids, flavonoids, anthocyanins, carotenoids, and other pigments, whereas liquid chromatography–tandem mass spectrometry (LC-MS/MS) enables structural identification through molecular mass determination and fragmentation analysis [71,84]. These methodologies are particularly valuable when evaluating extracts obtained through UAE, MAE, and NADES-based extraction, where multiple compound classes may be simultaneously recovered. In addition, nuclear magnetic resonance (NMR) can provide complementary structural information, especially for complex mixtures or novel compounds requiring further confirmation [85,86].

Although chromatographic techniques provide high sensitivity and selectivity, their application frequently requires extensive sample preparation, certified standards, and specialized instrumentation. As a result, analytical costs and methodological complexity remain important limitations for large-scale implementation Vicente-Zurdo, 2025 [66].

#### 8.1.2. Dietary Fiber and Prebiotic Fractions

Dietary fiber and prebiotic carbohydrates recovered from agro-industrial by-products require analytical approaches distinct from those commonly applied to low-molecular-weight metabolites. Total, soluble, and insoluble dietary fiber fractions are commonly quantified using standardized enzymatic–gravimetric methods established by the Association of Official Analytical Collaboration (AOAC), which remain the reference procedures for nutritional characterization and regulatory purposes [33,34]. In contrast, the characterization of prebiotic carbohydrates such as inulin, fructooligosaccharides, and other oligosaccharides frequently relies on chromatographic techniques including high-performance liquid chromatography (HPLC) and high-performance anion-exchange chromatography coupled with pulsed amperometric detection (HPAEC-PAD) [36,37,38,39].

Complementary spectroscopic techniques such as Fourier transform infrared spectroscopy (FTIR) and NMR have also been applied to evaluate polysaccharide structures, functional groups, and compositional modifications associated with extraction and processing conditions [86,87]. These analytical tools are particularly relevant for assessing fermentability, functional properties, and potential health benefits of fiber-rich fractions intended for food and nutraceutical applications.

#### 8.1.3. Lipids and Fatty Acids

Lipid-rich fractions recovered from agro-industrial by-products require analytical techniques capable of resolving highly complex mixtures. Gas chromatography–mass spectrometry (GC-MS) continues to be the reference methodology for fatty acid profiling following derivatization into fatty acid methyl esters, providing detailed information regarding saturated, monounsaturated, and polyunsaturated fatty acids [66,84].

NMR complements chromatographic analyses by providing information related to molecular structure, degree of unsaturation, sterol composition, and lipid authenticity [85,86]. More recently, lipidomics approaches based on high-resolution mass spectrometry (HRMS) have expanded analytical capabilities by enabling comprehensive characterization of complex lipid mixtures and facilitating the identification of biomarkers associated with specific biological activities [86].

Despite these advances, direct comparison among studies remains challenging because extraction conditions, raw materials, and analytical protocols vary considerably. Consequently, harmonization of analytical procedures and reporting standards remains necessary to improve reproducibility and facilitate technological assessment across different agro-industrial by-products.

#### 8.1.4. Proteins and Bioactive Peptides

Proteins and bioactive peptides recovered from agro-industrial by-products have attracted increasing attention because of their antioxidant, antimicrobial, antihypertensive, and immunomodulatory activities. Their characterization generally begins with electrophoretic techniques such as Sodium dodecyl sulfate-polyacrylamide gel electrophoresis (SDS-PAGE), which provide information regarding molecular weight distribution and protein integrity [71].

Subsequent identification of bioactive peptides typically relies on Reverse-phase high-performance liquid chromatography (RP-HPLC), Matrix-assisted laser desorption/ionization time-of-flight mass spectrometry (MALDI-TOF-MS), and LC-MS/MS. These techniques enable peptide purification, sequence determination, and structure–function analysis, facilitating the correlation of specific peptide sequences with biological activity [66]. This information is particularly relevant for the development of functional ingredients and nutraceutical products derived from agro-industrial by-products.

Because analytical requirements vary substantially according to the chemical nature of the recovered compounds, the most commonly employed analytical platforms and the information they provide are summarized in Table 4.

### 8.2. Foodomics Approaches and Emerging Analytical Trends

The increasing complexity of agro-industrial by-products has promoted the adoption of foodomics approaches that integrate advanced analytical platforms with computational and bioinformatics tools. Foodomics combines metabolomics, proteomics, lipidomics, and peptidomics to achieve comprehensive characterization of food-derived compounds and their biological effects [88].

Among these approaches, metabolomics has emerged as one of the most powerful strategies for investigating complex extracts. By integrating liquid chromatography–mass spectrometry (LC-MS), GC-MS, and NMR with multivariate statistical analyses, metabolomics enables both targeted and untargeted identification of metabolites, facilitating the discovery of biomarkers, assessment of extraction selectivity, and evaluation of changes induced by processing and storage conditions [86,88,89].

High-resolution mass spectrometry platforms, including Orbitrap and quadrupole time-of-flight (QTOF) systems, have further improved compound detection and structural elucidation capabilities. These technologies allow the identification of trace-level metabolites and provide detailed chemical fingerprints that support authentication, quality control, and process optimization [86,89]. In parallel, artificial intelligence-assisted data processing is increasingly being incorporated into foodomics workflows to improve pattern recognition and interpretation of large analytical datasets.

Biosensors also represent an emerging analytical alternative because they offer rapid, portable, and cost-effective detection of specific compounds, making them attractive for industrial monitoring and quality control applications [90]. Nevertheless, limited standardization and validation currently restrict their widespread implementation compared with established chromatographic and spectrometric platforms.

### 8.3. Current Challenges and Future Perspectives

Although significant advances have been achieved in the analytical characterization of bioactive compounds recovered from agro-industrial by-products, several challenges remain. Analytical methodologies frequently differ among studies, limiting direct comparison of extraction efficiencies, compound concentrations, and biological activities. Furthermore, many studies still report qualitative descriptions of bioactivity without incorporating quantitative metrics that facilitate comparison among compounds and extraction technologies.

Another important limitation concerns industrial scalability. While many green extraction technologies have demonstrated promising laboratory-scale performance, information regarding energy consumption, solvent recovery, process robustness, and production costs remains scarce. Consequently, analytical characterization should be integrated with techno-economic and sustainability assessments to support industrial implementation.

Beyond extraction yield, the industrial implementation of bioactive compound recovery processes requires a multidimensional evaluation framework that considers solvent consumption, energy demand, process robustness, scalability, regulatory acceptance, and environmental impact [5,8]. Comparative analyses of conventional and emerging extraction technologies indicate that ultrasound-assisted extraction (UAE) and pressurized liquid extraction (PLE) currently provide a favorable balance between extraction performance and industrial feasibility, whereas NADESs represent promising alternatives aligned with green chemistry and circular economy principles but still face challenges related to technological maturity and downstream processing [5,8,91]. Therefore, future studies should increasingly integrate Techno-economic assessment (TEA), Life cycle assessment (LCA), and Technology Readiness Level (TRL) evaluations to facilitate evidence-based selection of sustainable extraction strategies and support industrial-scale implementation of agro-industrial by-product valorization systems [5].

To facilitate a broader evaluation of extraction technologies employed for agro-industrial by-product valorization, Table 5 summarizes key technological, environmental, and industrial implementation criteria associated with conventional and green extraction approaches. Although the representative extraction performance data included in the table are derived from polyphenol recovery studies, the comparative sustainability, scalability, solvent requirements, and process evaluation criteria are broadly applicable to the recovery of other classes of bioactive compounds. It should be noted that extraction yields are highly dependent on biomass characteristics, target compounds, solvent systems, extraction parameters, and analytical methodologies employed.

Therefore, the reported values should be interpreted as representative examples rather than universally comparable performance indicators. Future efforts should prioritize standardized analytical workflows that combine sustainable extraction technologies with foodomics platforms, enabling more comprehensive characterization of recovered bioactive fractions and facilitating comparison among studies. Such integration would strengthen the development of circular biorefinery models and accelerate the conversion of agro-industrial by-products into high-value functional ingredients.

## 9. Industrial Applications of Agro-Industrial By-Products

The industrial relevance of agro-industrial by-products extends beyond the recovery of individual compounds, as these materials can serve as feedstocks for integrated biorefinery systems capable of generating multiple value-added products from a single residue stream. Such an approach maximizes resource utilization while reducing environmental burdens associated with waste disposal [45,49].

For instance, grape pomace generated during winemaking contains a complex mixture of polyphenols, dietary fiber, lipids, and pigments. Polyphenol-rich extracts can be utilized as natural antioxidants and preservatives, while the residual fibrous fraction may be incorporated into bakery products, functional foods, or biodegradable packaging materials. In parallel, grape seed oil rich in unsaturated fatty acids can be recovered for nutraceutical and cosmetic applications [48,94,95,96]. Similarly, olive pomace represents a multifunctional feedstock from which hydroxytyrosol-rich extracts, dietary fiber, and squalene-containing lipid fractions can be simultaneously obtained, yielding ingredients for food, pharmaceutical, and cosmetic industries [46,49,95].

Fruit processing by-products such as pomegranate peels, citrus peels, berry residues, and apple pomace also illustrate the versatility of agro-industrial residues. These materials provide polyphenols and natural pigments with antioxidant and coloring properties, while their structural carbohydrate fractions can be exploited as sources of dietary fiber and prebiotic ingredients. These bioactive compounds have been incorporated into meat products, dairy formulations, beverages, and edible films, simultaneously improving nutritional value, oxidative stability, and product functionality [25,39,40,97,98].

In addition, natural pigments recovered from fruit and vegetable residues are increasingly used as natural food colorants, nutraceutical ingredients, cosmetic additives, and components of active or intelligent packaging systems designed to monitor food quality and freshness [23,25]. However, pigment instability during processing and storage, particularly under variations in pH, temperature, oxygen exposure, and light, remains a major challenge for large-scale industrial implementation [23,83].

Lipid-rich residues generated during oilseed processing and vegetable-oil refining constitute another important valorization pathway. Rice bran, grape seeds, and deodorizer distillates contain phytosterols, tocopherols, phospholipids, and unsaturated fatty acids that are currently used in cholesterol-lowering foods, antioxidant formulations, lipid-based delivery systems, and high-value cosmetic products [46,47,48]. Likewise, protein-rich by-products from dairy, meat, fish, and plant industries provide substrates for the generation of bioactive peptides with antioxidant, antimicrobial, antihypertensive, and metabolic regulatory properties, supporting the development of next-generation functional ingredients and health-promoting formulations [52,53,58].

Collectively, these examples demonstrate that agro-industrial by-products should no longer be regarded as isolated sources of specific compounds but as multifunctional matrices capable of generating diverse ingredient portfolios. Their successful industrial implementation will increasingly depend on integrated biorefinery models that combine the sequential recovery of polyphenols, pigments, fibers, lipids, and peptides, thereby maximizing economic value while contributing to sustainability and circular bioeconomy goals [45,49,71].

## 10. Conclusions

Agro-industrial by-products represent valuable and multifunctional sources of bioactive compounds, including polyphenols, natural pigments, dietary fiber and prebiotics, lipids, proteins, and bioactive peptides. These residual streams should be regarded as complex biological matrices rather than isolated sources of individual compounds, since multiple bioactive compounds frequently coexist within the same raw material.

Despite their structural diversity, the recovery of these compounds presents common technological challenges related to extraction efficiency, compound stability, analytical characterization, process scalability, and industrial implementation. The increasing adoption of green extraction technologies, together with advances in analytical platforms and foodomics approaches, has expanded opportunities for their sustainable recovery and valorization.

The integration of different classes of bioactive compounds within a unified circular biorefinery framework connects raw material sources, extraction technologies, analytical characterization, biological functionality, and industrial applications. This holistic perspective highlights the potential of agro-industrial by-products to generate multiple value-added products through integrated recovery strategies.

Future research should prioritize the development of scalable extraction technologies, standardized analytical methodologies, and multidisciplinary evaluation frameworks integrating techno-economic, environmental, and technology readiness assessments. Such advances will facilitate the transition from laboratory-scale studies to industrial implementation, ultimately supporting sustainable and circular food production systems.

## Figures and Tables

**Table 1:** Dietary fiber and prebiotic index of agro-industrial by-products.

Agro-Industrial By-Product	Dietary Fiber (%)	Prebiotic Index
Cactus pear peel [40]	64.15 ± 2.05	0.33 [43]
Pineapple peel [40]	62.54 ± 3.2	0.32 [43]
Apple marc [39]	70.91 ± 1.30	ND
Carrot bagasse [45]	52.00 ± 0.81	0.42 [46]
Banana peel [46]	46.63 ± 2.7	0.28 [46]

**Table 2:** Representative lipid fractions recovered from agro-industrial by-products, their predominant compounds, reported bioactivities, applications, and typical extraction/recovery approaches.

Lipid Fraction	Source	Predominant Compounds	Reported Bioactivity	Application	Industrial Product	Extraction/Recovery Approach
Vegetable seed oils [45,46,47,48]	Grape seeds, mango kernels, olive pomace	Oleic acid, linoleic acid, α-linolenic acid	Antioxidant activity	Functional foods, cosmetics	Functional edible oils, cosmetic oils, omega-enriched formulations	Cold pressing, solvent extraction, SFE
Phytosterols [47,48]	Deodorizer distillates	β-sitosterol, campesterol, stigmasterol	Cholesterol lowering	Functional foods	Cholesterol-lowering spreads, fortified dairy products, nutraceutical supplements	Molecular distillation, fractionation
Tocopherols [45,48]	Rice bran, grape seed	α- and γ-tocopherol	Antioxidant	Nutraceuticals	Antioxidant supplements, oil stabilizers, vitamin E formulations	Distillation, solvent extraction
Phospholipids [46,48]	Oil refining streams	Phosphatidylcholine	Improved bioavailability	Lecithins, liposomes	Food emulsifiers, liposomal delivery systems, nutraceutical carriers	Degumming and purification
Fat-soluble vitamins [46,48]	Olive pomace	Squalene	Antioxidant, skin protection	Cosmetics, nutraceuticals	Anti-aging creams, skin-care formulations, nutraceutical capsules	Solvent extraction, molecular fractionation

**Table 3:** Summary of sensory evaluation studies of foods formulated with agro-industrial by-products.

Food Category	Food Product	Agro-Industrial By-Product	Incorporation Level	Sensory Method	Sensory Attributes and Statistical Significance	Main Findings
Functional foods [72]	Tomato purée	Grape pomace and grape skins	Particle size <125 µm and >125 µm	Descriptive sensory analysis (n = 9) and consumer acceptance (n = 95)	Texture, aroma, homogeneity, astringency (*p* < 0.05)	Smaller particles produced more homogeneous purées and traditional tomato flavor, whereas larger particles increased crunchiness and vegetal notes
Functional beverages [73]	Fermented wheat beverage	Fermented-extruded wheat; fruit residues (blueberry, raspberry, sea buckthorn, currant)	Wheat: 5, 10, 15 g/serving; fruit residues: 2.5, 5.0, 7.5 g/serving	10-point hedonic scale and facial emotion recognition (n = 50)	Acceptability, emotional responses (*p* < 0.05)	High acceptance scores (up to 9.5/10). Positive correlation between “happy” emotion and acceptance (r = 0.8525)
Snacks [74]	Extruded snacks	Defatted hazelnut flour and fruit residues	Hazelnut flour: 5–15%; fruit residues: 3–7%	9-point hedonic scale (n = 10)	Color, flavor, texture, overall acceptance (*p* < 0.05)	Higher incorporation increased product darkness, whereas moderate levels improved crunchiness and acceptability
Bakery products [75]	Cookies	Hibiscus sabdariffa residues	1.25%, 2.50%, 5%	7-point hedonic scale (n = 14)	Flavor, aroma, color, crunchiness, appearance (*p* < 0.05)	Moderate levels improved sensory scores; highest level reduced acceptance
Bakery products [76]	Cookies	Apple pomace	10% and 20% flour substitution	7-point hedonic scale (n = 30)	Fruity flavor, sweetness, texture, acidity (*p* < 0.05)	Apple pomace increased fruity flavor without affecting sweetness or texture
Cereal-based foods [77]	Pasta	Grape pomace	Enriched formulation	7-point hedonic scale (n = 14)	Color, flavor, texture (*p* < 0.05)	Slight color modification; minimal effect on flavor and texture
Cereal-based foods [78]	Pasta	Potato juice (fresh and dried)	Enriched formulation	10-point hedonic scale (n = 60)	Appearance, flavor, texture (*p* < 0.05)	Color identified as the main attribute influencing consumer perception
Cereal-based foods [79]	Pasta	Coconut flour and coconut milk residues	Coconut flour: 10–15%; coconut residues: 5–10%	7-point hedonic scale (n = 60)	Aroma, taste, texture, color (*p* < 0.05)	Minimal changes in taste and aroma; noticeable color modifications
Bakery products [80]	Muffins	Cauliflower by-product flour	10%, 20%, 30% substitution	9-point hedonic scale (n = 10)	Appearance, flavor, texture, overall acceptance (*p* < 0.05)	Moderate substitution maintained sensory quality, whereas higher levels reduced acceptability
Meat products [81]	Beef burgers	Flaxseed and flaxseed by-products	0% to 100%	9-point hedonic scale (n = 15)	Flavor, aroma, appearance, texture (*p* < 0.05)	The incorporation of 50% maintained acceptable sensory scores; higher levels negatively affected flavor and texture
Dairy products [82]	Goat cheese	Citrus pulp (animal diet supplementation)	Dietary supplementation	Descriptive sensory analysis (n = 10) and consumer acceptance (n = 80)	Odor attributes (milk, butter, sweet, toffee, nuts, goat aroma) (*p* < 0.05)	Similar sensory profiles dominated by buttery and acidic notes

**Table 4:** Main analytical tools used for the characterization of bioactive compounds in agro-industrial by-products.

Bioactive Compound Class	Main Analytical Techniques	Main Information Obtained	Typical Green Extraction Technologies Associated	Representative References
Polyphenols	HPLC-DAD, LC-MS/MS, NMR	Identification, quantification and structural elucidation of phenolic compounds	UAE, MAE, PLE, NADESs	[16,71,84,85]
Natural pigments	UV-Vis, HPLC-DAD, LC-MS/MS	Pigment profiling, stability assessment and degradation products	UAE, MAE, SFE	[71,84]
Dietary fiber and prebiotics	AOAC enzymatic–gravimetric methods, HPLC, HPAEC-PAD, FTIR, NMR	Fiber composition, oligosaccharide profile, structural characterization and fermentability assessment	UAE, MAE, Enzymatic extraction	[33,36,39,85,87]
Lipids and fatty acids	GC-MS, NMR, Lipidomics	Fatty acid composition, sterol profile, degree of unsaturation and lipid fingerprinting	SFE, PLE	[66,84,85,86]
Proteins and bioactive peptides	SDS-PAGE, RP-HPLC, MALDI-TOF-MS, LC-MS/MS	Molecular characterization, peptide identification and structure–activity relationships	UAE, MAE, Enzymatic extraction	[66,71,86]
Complex extracts	HRMS, Metabolomics and Foodomics platforms	Global metabolic profiling, biomarker discovery, authentication and extraction selectivity assessment	UAE, MAE, SFE, NADESs	[86,88,89]
Industrial quality control	FTIR, Biosensors	Rapid screening, process monitoring and routine quality control	Applicable across extraction technologies	[87,90]

Note: Quantitative performance indicators reported for green extraction technologies are often influenced by biomass characteristics, extraction conditions, process scale, and methodological assumptions, which may limit direct comparison among studies.

**Table 5:** Comparative assessment of conventional and green extraction technologies used in the recovery of bioactive compounds from agro-industrial by-products.

Extraction Technology	Polyphenol Yield Range (mg GAE/g DW)	Solvent Consumption	Energy Demand	Industrial Scalability	Green Chemistry Alignment	Circular Economy Contribution	Main Limitations
Conventional solvent extraction (CS/E) [8,92]	10–45	High (20–100 mL/g biomass)	Moderate	High	Low–Moderate	Enables valorization of agro-industrial by-products but generates significant solvent waste and requires solvent recovery operations	Long extraction times, high solvent consumption, lower selectivity, environmental burden
Ultrasound-assisted extraction (UAE) [8,92,93]	20–70	Low–Moderate (5–30 mL/g biomass)	Low–Moderate	High	High	Reduces solvent usage and processing time, improving resource efficiency and facilitating by-product valorization	Process optimization required; cavitation may affect sensitive compounds under extreme conditions
Microwave-assisted extraction (MAE) [8,92]	25–80	Low–Moderate	Moderate	Moderate–High	High	Improves extraction efficiency while reducing extraction time and solvent requirements	Potential degradation of thermolabile compounds; scale-up challenges remain
Pressurized liquid extraction (PLE) [5,8]	30–90	Low	Moderate	High	High	Compatible with biorefinery concepts through efficient utilization of water and ethanol under controlled conditions	Higher equipment costs and operation under elevated pressure
Supercritical fluid extraction (SFE-CO_2_ + ethanol) [8]	15–65	Very low organic solvent use	High	Moderate–High	Very High	Allows solvent recycling and production of high-purity extracts with minimal environmental impact	High capital investment; limited recovery of highly polar phenolics without co-solvents
Natural deep eutectic solvents (NADESs) [5,91]	40–100	Very low toxicity solvents	Low–Moderate	Emerging	Very High	Strong compatibility with circular bioeconomy principles due to biodegradable and renewable solvent components	High viscosity, challenging downstream purification, limited industrial implementation, regulatory uncertainty

Abbreviations: GAE, gallic acid equivalents; DW, dry weight. Note: Representative extraction yield ranges correspond to selected polyphenol recovery studies and are provided for illustrative purposes only. Reported values may vary substantially depending on biomass composition, target compounds, solvent selection, extraction conditions, process parameters, and analytical methodologies. Consequently, direct quantitative comparisons among studies should be interpreted with caution.

## Data Availability

No new data were created or analyzed in this study. Data sharing is not applicable to this article.

## Supplementary Materials

The following supporting information can be downloaded at: https://www.mdpi.com/article/10.3390/foods15132406/s1, Table S1: Representative experimental studies on polyphenol recovery from agro-industrial by-products using conventional and green extraction technologies.

## Abbreviations

The following abbreviations are used in this manuscript:

AACC	American Association of Cereal Chemists
ABTS	2,2′-azinobis(3-ethylbenzothiazoline-6-sulfonic acid)
AOAC	Association of Official Analytical Collaboration
CE	Circular economy
CSE	Conventional solvent extraction
DPPH	2,2-diphenyl-1-picrylhydrazyl
DW	Dry weight
FAO	Food and Agriculture Organization
FTIR	Fourier transform infrared spectroscopy
GAE	Gallic acid equivalents
GC-MS	Gas chromatography–mass spectrometry
HPAEC-PAD	High-performance anion-exchange chromatography with pulsed amperometric detection
HPLC	High-performance liquid chromatography
HPLC-DAD	High-performance liquid chromatography with diode array detection
HRMS	High-resolution mass spectrometry
LCA	Life cycle assessment
LC-MS	Liquid chromatography–mass spectrometry
LC-MS/MS	Liquid chromatography–tandem mass spectrometry
MAE	Microwave-assisted extraction
MALDI-TOF-MS	Matrix-assisted laser desorption/ionization time-of-flight mass spectrometry
NADESs	Natural deep eutectic solvents
NMR	Nuclear magnetic resonance
PLE	Pressurized liquid extraction
PRISMA	Preferred Reporting Items for Systematic Reviews and Meta-Analyses
RP-HPLC	Reverse-phase high-performance liquid chromatography
SCFAs	Short-chain fatty acids
SDS-PAGE	Sodium dodecyl sulfate–polyacrylamide gel electrophoresis
SFE	Supercritical fluid extraction
TEA	Techno-economic assessment
TRL	Technology readiness level
UAE	Ultrasound-assisted extraction
UV–Vis	Ultraviolet–visible spectroscopy
