# Supplementary material for: Bioactive Compounds from Agro-Industrial By-Products: Green Recovery Technologies, Analytical Characterization, and Industrial Applications
Source: Foods. 2026 Jul 7;15(13):2406. doi: 10.3390/foods15132406 (PMC13360740; doi:10.3390/foods15132406)
Supplement: Supplementary file 1 [file foods-15-02406-s001.zip › foods-4313293-supplementary.pdf]

Table S1. Representative experimental studies on polyphenol recovery from agro-industrial by-products using conventional and green extraction technologies.

Agro-industrial by-product	Main polyphenols recovered	Extraction technology	Extraction conditions	Polyphenol yield (mg GAE/g DW)	Bioactivity assay
Grape pomace [17]	Catechin, epicatechin, quercetin, resveratrol	Enzyme-assisted extraction followed by solvent extraction	Cellulase-assisted extraction	41.05 ± 1.07 mg/g DW	Total polyphenol content; antioxidant evaluation
Olive pomace [18]	Hydroxytyrosol, oleuropein derivatives, secoiridoids	Pressurized Liquid Extraction (PLE)	Ethanol-water mixtures under pressure	1659 mg/kg DW (≈1.66 mg/g DW phenolic extract)	HPLC-DAD-ESI-TOF/MS characterization
Pomegranate peel [19]	Punicalagin, ellagic acid, gallic acid	Pressurized Liquid Extraction (PLE)	Optimized temperature and solvent conditions	264.3 mg GAE/g DW	Antioxidant activity
Pomegranate peel [19]	Punicalagin	Microwave-Assisted Extraction (MAE)	50% ethanol, optimized microwave power	Punicalagin: 143.64 mg/g DW	Radical scavenging activity (94.91%)
Pomegranate peel [93]	Punicalagin, ellagic acid	Ultrasound-Assisted Extraction (UAE)	50% aqueous ethanol	101.6 mg GAE/g DW (10,159 mg GAE/100 g DW)	DPPH, ABTS, CUPRAC
Apple pomace [20]	Chlorogenic acid, phloridzin, quercetin glycosides	Ultrasound-Assisted Extraction (UAE)	20 min, 90 °C, 50% ultrasound amplitude	3.61 ± 0.57 mg GAE/g DW	DPPH antioxidant assay
Berry processing residues (blackberry residues) [21]	Anthocyanins, flavonoids, phenolic acids	Ultrasound-Assisted Extraction (UAE)	91% amplitude, 15 min	12.01 mg GAE/g DW (1201.23 mg GAE/100 g DW)	ABTS and DPPH antioxidant assays

Reported values correspond to experimental studies employing different extraction conditions, solvents, analytical methodologies, and biomass characteristics. Therefore, direct quantitative comparisons among studies should be interpreted cautiously.
